# Supplementary material for: High-throughput phenomics of global ant biodiversity
Source: Nat Methods. 2026 Mar 5;23(3):663–72. doi: 10.1038/s41592-026-03005-0 (PMC12982114; doi:10.1038/s41592-026-03005-0)
Supplement: Supplementary file 1 — Reporting Summary [file 41592_2026_3005_MOESM1_ESM.pdf]

nature portfolio

Corresponding author(s): Julian Katzke, Francisco Hita Garcia, Evan P. Economo, Thomas van de Kamp

Last updated by author(s): May 15, 2025

Reporting Summary

Nature Portfolio wishes to improve the reproducibility of the work that we publish. This form provides structure for consistency and transparency in reporting. For further information on Nature Portfolio policies, see our Editorial Policies and the Editorial Policy Checklist.

Statistics

For all statistical analyses, confirm that the following items are present in the figure legend, table legend, main text, or Methods section.

n/a	Confirmed	
☒	☐	The exact sample size (*n*) for each experimental group/condition, given as a discrete number and unit of measurement
☒	☐	A statement on whether measurements were taken from distinct samples or whether the same sample was measured repeatedly
☒	☐	The statistical test(s) used AND whether they are one- or two-sided<br>*Only common tests should be described solely by name; describe more complex techniques in the Methods section.*
☒	☐	A description of all covariates tested
☒	☐	A description of any assumptions or corrections, such as tests of normality and adjustment for multiple comparisons
☒	☐	A full description of the statistical parameters including central tendency (e.g. means) or other basic estimates (e.g. regression coefficient) AND variation (e.g. standard deviation) or associated estimates of uncertainty (e.g. confidence intervals)
☒	☐	For null hypothesis testing, the test statistic (e.g. *F*, *t*, *r*) with confidence intervals, effect sizes, degrees of freedom and *P* value noted<br>*Give P values as exact values whenever suitable.*
☒	☐	For Bayesian analysis, information on the choice of priors and Markov chain Monte Carlo settings
☒	☐	For hierarchical and complex designs, identification of the appropriate level for tests and full reporting of outcomes
☒	☐	Estimates of effect sizes (e.g. Cohen's *d*, Pearson's *r*), indicating how they were calculated

Our web collection on statistics for biologists contains articles on many of the points above.

Software and code

Policy information about availability of computer code

Data collection	We employed the control system concert 0.31.0 and the UFO 0.16 framework for data acquisition and online reconstruction of tomographic slices. Final reconstruction of tomographic data was done with tofu 0.9. All software packages are referenced in the Methods section.
Data analysis	Data were analyzed using R 4.4.2, Python 3.10.12, numpy 1.24.3 , Simple-ITK 2.3.1, SciPy 1.11.4, Biomedisa 23.09.1, Paraview 2.12, Fiji 2.15.1, Amira 2020.2, 3D Slicer 5.8.1 & CINEMA 4D R20. Code available at https://github.com/julesforfools/Antscan.

For manuscripts utilizing custom algorithms or software that are central to the research but not yet described in published literature, software must be made available to editors and reviewers. We strongly encourage code deposition in a community repository (e.g. GitHub). See the Nature Portfolio guidelines for submitting code & software for further information.

Data

Policy information about availability of data

All manuscripts must include a data availability statement. This statement should provide the following information, where applicable:

- Accession codes, unique identifiers, or web links for publicly available datasets
- A description of any restrictions on data availability
- For clinical datasets or third party data, please ensure that the statement adheres to our policy

All processed Antscan tomograms are stored at the Large Scale Data Facility (LSDF) of KIT’s Scientific Computing Center (SCC) and integrated into Biomedisa for public access (https://biomedisa.info/antscan/). Additionally, all datasets are archived at the RADAR4KIT repository (https://radar.kit.edu/radar/en/search?

query=antscan). Both versions of the database and all metadata and DOIs linked to datasets are accessible via https://antscan.info . All metadata is also presented in the supplemental information to this publication. Raw X-ray projections and all other image files are further stored at KIT and will be made accessible upon reasonable request.

Human research participants

Policy information about studies involving human research participants and Sex and Gender in Research.

Reporting on sex and gender	n/a
Population characteristics	n/a
Recruitment	n/a
Ethics oversight	n/a

Note that full information on the approval of the study protocol must also be provided in the manuscript.

Field-specific reporting

Please select the one below that is the best fit for your research. If you are not sure, read the appropriate sections before making your selection.

☒ Life sciences ☐ Behavioural & social sciences ☐ Ecological, evolutionary & environmental sciences

For a reference copy of the document with all sections, see nature.com/documents/nr-reporting-summary-flat.pdf

Life sciences study design

All studies must disclose on these points even when the disclosure is negative.

Sample size	Our study does not deal with experimental series of similar samples, but presents a collection of diverse specimens. Therefore no sample size calculation was required.
Data exclusions	No data were excluded from the analyses.
Replication	The reproducibility of the experimental results is guaranteed by the description of the methods in the manuscript and by the data and code provided
Randomization	The study does not include experimental series but is based on individual specimens.
Blinding	This paper does not deal with experimental series of similar samples, which would require statistical analysis. Blinding was therefore not applicable here.

Reporting for specific materials, systems and methods

We require information from authors about some types of materials, experimental systems and methods used in many studies. Here, indicate whether each material, system or method listed is relevant to your study. If you are not sure if a list item applies to your research, read the appropriate section before selecting a response.

Materials & experimental systems

n/a	Involved in the study	
☒	☐	Antibodies
☒	☐	Eukaryotic cell lines
☒	☐	Palaeontology and archaeology
☐	☒	Animals and other organisms
☒	☐	Clinical data
☒	☐	Dual use research of concern

Methods

n/a	Involved in the study	
☒	☐	ChIP-seq
☒	☐	Flow cytometry
☒	☐	MRI-based neuroimaging

Animals and other research organisms

Policy information about studies involving animals; ARRIVE guidelines recommended for reporting animal research, and Sex and Gender in Research

Laboratory animals	The study does not involve laboratory animals
Wild animals	The study does not involve wild animals.
Reporting on sex	The findings themselves are not sex-specific. However, while we have included male ants, a natural female bias arises because all ant workers are female. The sex of each specimen is specified in the metadata accompanying each tomogram and listed in the Supplementary Data.
Field-collected samples	No new samples were collected for this study. All specimens originate from standing insect collections as detailed in the metadata accompanying each tomogram and listed in the Supplementary Data.
Ethics oversight	All study specimens are ethanol-fixed insects from standing insect collections, which do not require ethical approval or guidance. All export and import of specimens was conducted according to national and international guidelines through permits and institutional collaborations.

Note that full information on the approval of the study protocol must also be provided in the manuscript.
